# Prevalence of HIV/AIDS and Prediction of Future Trends in North-west Region of India: A six-year ICTC-based Study

**DOI:** 10.4103/0970-0218.55286

**Published:** 2009-07

**Authors:** Nitya Vyas, Saroj Hooja, Parul Sinha, Anuj Mathur, Anita Singhal, Leela Vyas

**Affiliations:** Department of Microbiology and Immunology, Sawai Man Singh Medical College, Jaipur, India

**Keywords:** HIV, prevalence, prediction, India

## Abstract

**Background:**

The study was conducted to analyze previous six-year prevalence data of HIV infection in the Northwest region of India and predict future trends for a couple of years.

**Objectives:**

The study was conducted to aid SACS and NACO to plan and arrange resources for the future scenario.

**Materials and Methods:**

All the attendees of ICTC, Jaipur, from January 2002 to December 2007 were included and variables like age, sex, marital status, occupation, place of residence, pattern of risk behavior and HIV serostatus were studied. As per the strategy and policy prescribed by NACO, tests (E/R/S) were performed on the serum samples. Data was collected; compiled and analyzed using standard statistical methods. Future trends of HIV-prevalence in north-west India were anticipated.

**Results:**

The overall positivity rates among attendees of ICTC, were found to be 12.2% (386/3161), 11.8% (519/4381), 11.1% (649/5867), 13% (908/6983), 14% (1385/9911) and 17.34% (1756/10133) in the years 2002, 2003, 2004, 2005, 2006 and 2007 respectively. Future trends for the next couple of years depict further increase in prevalence without any plateau.

**Conclusion:**

Epidemiological studies should be carried out in various settings to understand the role and complex relations of innumerable behavioral, social and demographic factors, which will help, interrupt and control the transmission of HIV/ AIDS.

## Introduction

The Human Immunodeficiency virus (HIV)/Acquired Immunodeficiency syndrome (AIDS) epidemic has devastated many individuals, families and communities. As the epidemic evolves further, rates will continue to rise in communities and nations where poverty, social inequalities, and weak health infrastructures facilitate spread of the virus. The estimate of 5.7 million HIV-infected people in India (in year 2006), as compared with 5.5 million in South Africa, has captured wide attention. However, it remains uncertain if India has more infected people than any other country. The epidemiologic data for India (number of infected persons range from 3.4 million to 9.4 million) is far less precise than for South Africa (4.9 million to 6.1 million),(1) as the estimate for India is based primarily on anonymous testing data from public clinics for antenatal care, patients in high-risk groups or with sexually transmitted infections (STDs).(2) Although the number of surveillance sites is expanding, the data may still be skewed and inadequate(3) as one per cent increase in the HIV prevalence in adults would result in an additional five million infected people(4) i.e. small changes in prevalence could translate to large absolute numbers of infected individuals and that's why the true prevalence is still disputed.

National AIDS Control Organization (NACO) and Ministry of Health and Family Welfare, Government of India in 2005 declared six states (Andhra Pradesh, Karnataka, Maharashtra, Manipur, Nagaland, and Tamil Nadu) as high prevalence areas (defined by a rate of HIV positivity of more than one percent among women visiting pre-natal clinics and a rate of more than five per cent among patients visiting clinics for STDs) Moderate prevalence (rate of HIV positivity of less than one per cent among women visiting prenatal clinics and a rate of more than five per cent among patients visiting clinics for STDs) was found in Gujarat, Goa, and the Union Territory of Pondicherry.(1)

It is worrisome that only an estimated 10 to 20% of those infected with HIV know that they are infected, which impedes treatment and prevention efforts.(1) To cope with these challenges, new models of care and cost-effective health care delivery systems are needed with proper understanding of the HIV/AIDS epidemiology in a particular region especially with regards to various socio demographic factors, level of awareness as well as pattern of risk behavior of the population, because till date, the most effective approaches available are awareness generation and lifestyle changes. The Integrated Counseling and Testing Centre (ICTC) is an entry point to care, which provides people with an opportunity to learn and accept their serostatus in a confidential environment.(5) The data generated in the ICTC may provide important clues to understand the epidemiology of the disease in a particular region.(5)

The present study was conducted on attendees of ICTC Department of Microbiology and Immunology of Sawai Man Singh Medical College, Jaipur (Rajasthan), to analyze previous six year prevalence data and predict the future trends for a couple of years. As this Institute is the only apex hospital in the region, the information gathered from attendees of this centre may throw light on the epidemiology of HIV transmission in this area.

## Materials and Methods

The study included all the attendees of ICTC from January 2002 to December 2007 coming either voluntarily or being referred from various departments of the Hospital. All the essential information was collected from the attendees by interviewing them. The variables studied included age, sex, marital status, occupation, place of residence, pattern of risk behavior and HIV serostatus.

Following the guidelines of NACO, the counselor of the ICTC interviewed the attendees under strict confidentiality. After pre- test counseling and obtaining consent of the attendees, laboratory technician in the Department of Microbiology collected their blood samples. As per the strategy and policy prescribed by NACO, tests (E/R/S) were performed on the serum samples. Data was collected; compiled and analyzed using standard statistical methods and future trends of HIV prevalence in north- west India is also anticipated.

## Results

**Figure 1:**
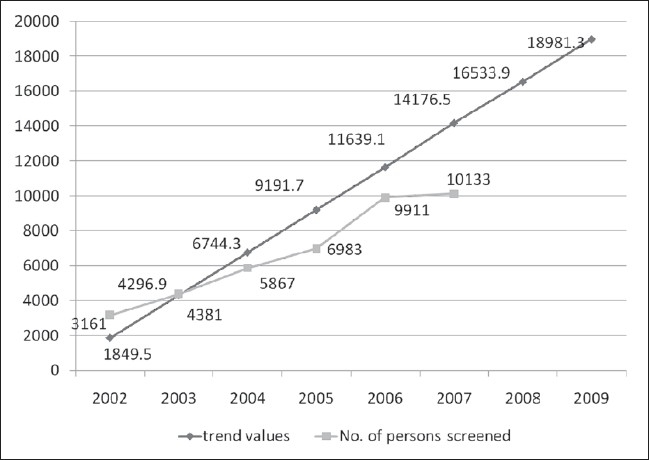
Year wise distribution of ICTC attendees and the trend values

There was a gradual increase in the number of people getting tested at ICTC, Jaipur each year; 3161 (in 2002), 4381 (in 2003), 5867 (in 2004), 6983 (in 2005), 9911 (in 2006) and10, 133 (in 2007) as shown in Figure 1.

**Figure 2:**
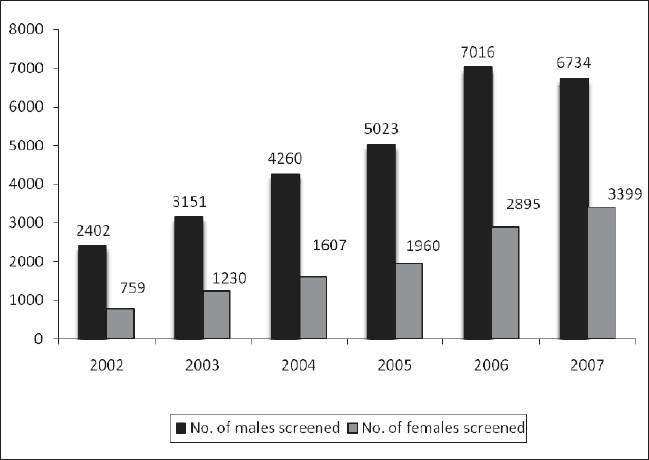
Sex wise distribution of ICTC attendees

There was a large difference in male to female testing ratio 3.1:1, 2.5:1, 2.6:1, 2.5:1, 2.4:1, and 1.98:1 in the years 2002, 2003, 2004, 2005, 2006 and 2007 respectively as shown in Figure 2.

**Figure 3:**
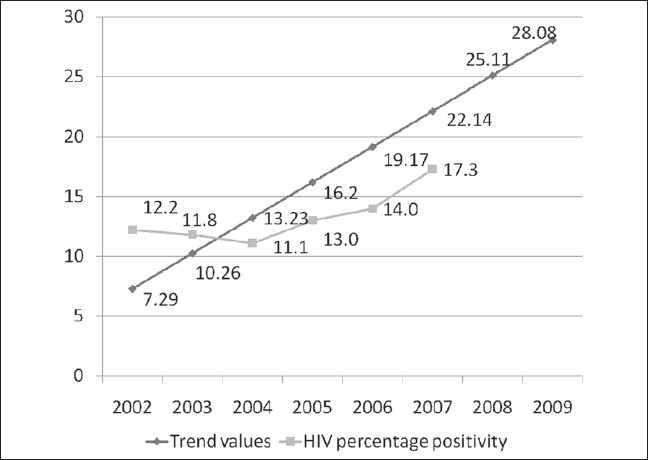
Year wise distribution of HIV seropositives and the trend values

**Figure 4:**
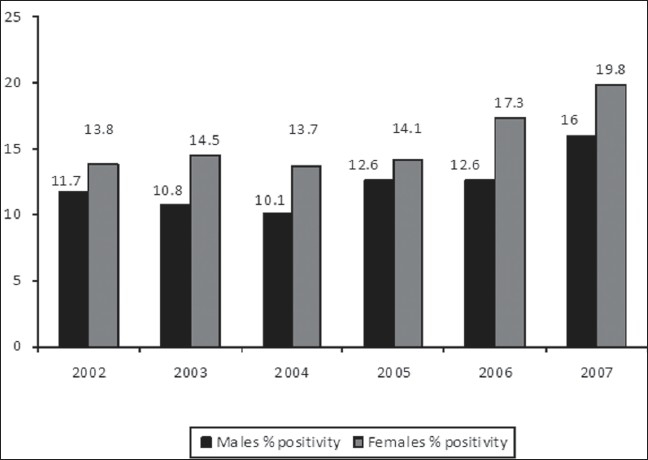
Sex wise distribution of HIV seropositives

Overall positivity rates among attendees of ICTC, were found to be 12.2% (386/3161), 11.8% (519/4381), 11.1% (649/5867), 13% (908/6983), 14 %(1385/9911) and 17.34% (1756/10133) in the years 2002, 2003, 2004, 2005, 2006 and 2007 respectively as shown in Figure 3 and their sex wise distribution is shown in Figure 4. Age and gender-wise distribution of HIV seropositivity among the attendees is shown in Table 1. Majority of seropositives belong to the age bracket of 15-49 years (87.0%, 90.5%, 89.2%, 86.67%, 87.07% and 85.99% in the years 2002, 2003, 2004, 2005, 2006 and 2007 respectively).

**Table 1:** Age and sex wise positivity rates among ICTC attendees

Year	Age in yrs	Male		Female		Total	
				
		No. screened	% HIV+ve (No.)	No. screened	% HIV+ve (No.)	No. screened	% HIV+ve (No.)
2002	<15	212	11.8 (25)	127	4.7 (06)	339	9.1 (31)
	15-49	1841	13.1 (242)	568	17.2 (98)	2409	14.1 (340)
	>50	349	4.0 (14)	64	1.6 (01)	413	3.8 (15)
	Total	2402	11.7 (281)	759	13.8 (105)	3161	12.2 (386)
2003	<15	255	6.3 (16)	154	4.5 (07)	409	5.6 (23)
	15-49	2416	12.7 (309)	954	17.5 (167)	3370	14.1 (476)
	>50	480	3.3 (16)	122	3.3 (04)	602	3.3 (20)
	Total	3151	10.8 (341)	1230	14.5 (178)	4381	11.8 (519)
2004	<15	315	9.2 (29)	177	6.8 (12)	492	8.3 (41)
	15-49	3274	11.7 (384)	1256	16.0 (201)	4516	12.9 (585)
	>50	671	2.4 (16)	174	4 (07)	845	2.7 (23)
	Not specified	08	00	06	00	14	00
	Total	6548	11.7 (768)	2512	16.0 (402)	9046	12.9 (1170)
2005	<15	473	11 (52)	231	8.6 (20)	704	10.2 (72)
	15-49	3808+9E	15.7 (600)+1E	1768	15.2 (269)	6049	14.3 (869)+1E
	>50	742	4.2 (31)	192	4.2 (08)	934	4.2 (39)
	Total	5023+9E	12.5 (631)+1E	1960	14.1 (277)	6983	13.0 (908)+1E
2006	<15	585	12.3 (72)	343	11.4 (39)	928	12.0 (111)
	15-49	5915+14E	13.0 (770)+3E	2274	19.7 (449)	7604	16.0 (1219)+3E
	>50	1101	3.8 (42)	278	4.7 (13)	1379	4.0 (55)
	Total	7016+14E	12.6 (884)+3E	2895	17.3 (501)	9911	14.0 (1385)+3E
2007	<15	729	10.1 (74)	401	13.4 (54)	1130	11.3 (128)
	15-49	4972+7E	8.2 (408)+1E	2614	22.5 (590)	7586	9.6 (732)+1E
	>50	1033	7.3 (75)	384	7.3 (28)	1417	7.3 (103)
	Total	6734+7E	16.0 (1084)+1E	3399	19.8 (672)	10133	17.3 (1756)+1E

E: Eunuchs; +ve: Positive

The distribution of HIV seropositives by their marital status shows that among males, majority (84.8% in 2002, 95.1% in 2003, 95.1% in 2004, 96.2% in 2005, 94.2% in 2006 and 93.36% in 2007) belong to the married group. Same pattern was observed among females also; 81.8%, 83.0%, 81.7%, 83.3%, 86.1% and 79.20% in the years 2002, 2003, 2004, 2005, 2006 and 2007 respectively.

Mother to child transmission (Perinatal transmission) was observed in 9.1% (931/339), 5.6 % (23/409), 8.3% (41/492), 10.2% (72/704), 12.0% (111/928) and 11.3% (128/1756) in years 2002, 2003, 2004, 2005, 2006 and 2007 respectively, as shown in Table 1.

The occupation level and HIV serostatus of the attendees show that among males, majority of seropositives were drivers, agriculture or unskilled workers and businessmen whereas among females, majority were housewives, (percentages are mentioned in the Table 2).

**Table 2:** Distribution of HIV seropositives by occupation

Occupation	2002 % (No.)	2003 % (No.)	2004 % (No.)	2005 % (No.)	2006 % (No.)	2007 % (No.)
Unskilled	9.5 (37)	8.4(44)	8.9 (58)	11.1 (101)	12.7 (177)	12.5 (221)
Skilled	4.9 (19)	3.6 (19)	4.7 (31)	5.0 (46)	3.6 (51)	4.6 (82)
Tile worker	2.0 (08)	3.8 (20)	2.6 (17)	3.9 (36)	0.7 (11)	1.6 (29)
Businessman	11.3 (44)	6.9 (36)	8.6 (56)	11.0 (100)	8.8 (123)	6.2 (110)
Service	15.2 (59)	9.6 (50)	4.9 (32)	2.8 (26)	3.6 (51)	6.8 (120)
Farmer	6.7 (26)	8.4 (44)	12.7 (83)	11.9 (109)	12.3 (171)	13.1 (230)
Driver	13.4 (52)	15.6 (81)	17.4 (113)	14.5 (132)	13.3 (185)	9.7 (172)
Housewife	25.6 (99)	32.9 (171)	31.8 (207)	28.2 (257)	33.0 (459)	33.6 (591)
Others	10.8 (42)	10.4 (54)	8.0 (52)	11.2 (102)	11.5 (160)	11.4 (201)
Total	(386)	(519)	(649)	(908+1E)	(1385+3E)	(1756+1E)

E: Eunuchs

The pattern of risk behaviour and HIV serostatus of the attendees [Table 3] show that for both males and females, seropositivity is maximum with those giving history of high risk sexual contact; for males 61.9% (174/281), 62.4% (213/341), 61.7% (265/429), 52.6% (332/631), 73.6% (651/884), 83.1% (901/1084) in years 2002, 2003, 2004, 2005, 2006 and 2007 respectively and for females; 66.6% (70/105), 61.2% (109/178), 76.8% (169/220), 70.0% (194/277), 81.6% (409/501) and 86.16% (579/672) in years 2002, 2003, 2004, 2005, 2006 and 2007 respectively.

**Table 3:** Pattern of risk behavior among HIV seropositives

Route of transmission	2002 % (No.)	2003 % (No.)	2004 % (No.)	2005 % (No.)	2006 % (No.)	2007 % (No.)
HRSC	63.2 (244)	62.0 (322)	66.8 (434)	57.9 (526)	76.8 (1064)	84.2 (1480)
BT	0.5 (02)	1.9 (10)	1.5 (10)	1.4 (13)	0.7 (10)	0.6 (11)
PN	8.0 (31)	4.4 (23)	6.3 (41)	7.9 (72)	7.9 (110)	7.2 (128)
IVDU	0.5 (02)	0.3 (02)	0.4 (03)	0.3 (03)	0.2 (03)	0(0)
HNA	26.4 (102)	31.2 (162)	24.8 (161)	32.3 (294)	14.2 (198)	7.8 (137)
Homosexual	0	0	0	0.1 (01)	0.2 (03)	0.05 (01)
Total	386	519	649	908+1E	1385+3E	1756+1E

E: Eunuchs; HRSC: High risk sexual contact; BT: Blood transfusion; PN: Perinatal; IVDU: Intravenous Drug Use; HNA: History not available

Areas like Jhunjhunu and Sikar came out to be the hot spots in comparison to other regions of Rajasthan. Only 46 seropositive cases gave history of previous blood transfusion over the period from 2002 to 2007. Only 13 seropositive cases were among intravenous drug users (IDUs) category over these six-year period. Out of around 30 eunuchs tested over the study period, 16.66% (5/30) came out to be seropositive for HIV (one in 2005 and three in 2006 and one in 2007).

## Discussion

The attendees of ICTC have shown a significant increase from 2402 (in year 2002) to 10133 (in year 2007). There was also an increase in the percentage of female patients being tested at ICTC from 24.0% (759/3161) in the year 2002 to 33.5% (3399/10133) in 2007. This may be attributed to either increased awareness about the disease; lesser stigma associated with it nowadays, expanded coverage of testing or probably due to more number of people feeling the need to get tested just because of availability of anti retroviral therapy (ART). But the large difference in male to female testing ratio (3.1:1, 2.5:1, 2.6:1, 2.5:1, 2.4:1 and 1.98:1 in the years 2002, 2003, 2004, 2005, 2006 and 2007 respectively) shows that females are still not availing of the medical facilities as much as males. Considering the national data based on information collected from sentinel surveillance sites, women are less likely to visit an antenatal clinic/testing centers if: they are older, have high parity, are illiterate, or are poor.(6) Programs for increasing female attendance in the health care centers should be carried out.

Although lesser number of females were tested in the present study, but positivity rates were found to be higher among them. This is because females are generally tested, after the diagnosis of their husbands and that is why the probability of seropositivity among them was found to be higher. As epidemics of HIV infections fueled largely by heterosexual transmission have developed in resource-poor countries, the impact of pandemic on women is rising, even in countries where other routes of transmission are more prevalent.

The present data depicting an overall increase in prevalence over these six year period from 12.2% (386/3161) in the year 2002 to 17.3% (1756/10133) in 2007 indicates that the upward trend is unabated in India without any sign of plateau. Also, one cannot deny the fact that seropositivity usually increases in initial stages when the program is expanding as it tests the old cases also. Secondly, with ART being used, there is increase in prevalence due to increased longevity of the patients. But it does not correlate with the data, which suggests slowing of any overall increase in prevalence.(1)

It was also observed that even a slight increase in the percentage positivity indicates a large increase in the total number of people infected by HIV (from 386 in the year 2002 to 1756 in 2007). The positivity rates of the year 2007 (17.34%), in our study were comparable to the study done in VCTC, Darjeeling, West Bengal(5) (17.06%) in 2003 but lower than that reported from VCTC, Jodhpur, Rajasthan (22.0%) in 2003-04.(7)

The findings in the present study on age distribution of HIV positives (ranging from 85.99% to 90.55% in age group of 15-49 years) corroborates with our national figure, where it is observed that most of the cases (about 89%) occurred among sexually active persons aged 20-49 years.(8,9)

Majority of the HIV seropositives belong to the married group irrespective of their sexes. Those who are unmarried will soon enter their reproductive lives and ultimately the risk of parent to child transmission will further increase. The peri-natal transmission in the present study was found to be ranging from 5.6% to 12.0% over the period of these six years. This was found to be little higher than that reported by Joardar *et al*.(5) (2.63%). Mother-to-child transmission can occur in utero, intrapartum, or postpartum during breast-feeding. In industrialized countries, advances in the prevention of mother-to-child transmission have substantially reduced the number of children who acquire HIV.(10) In India, poor access to health care system, ignorance of the masses, financial constraints and NACO policy till recently of not screening all the antenatal mothers unless the prevalence rates exceed one percent in the area may lead to increasing pediatric- HIV infections in coming years.(11) In Rajasthan although Prevention of parent to child transmission (PPTCT) programme has been running since a couple of years; the benefits, however, may not be evident for years to come.

As depicted in our study, businessmen staying away from their families may indulge in some risk behaviors that favours HIV transmission. Commercial sex and substance abuse are firmly entrenched in the socio- cultural milieu of the trucking industry in India and are a part of their daily lives.(5) Truckers and migrants may become infected while away and infect their wives when they return home. The transmission of HIV may stop there. Such “truncated epidemics” are characteristic of rural areas of India and Pakistan as men may not engage in high-risk behaviors when they are close to home.(1) So it may be presumed that the population in the study area is vulnerable for a rapid spread of infection due to its geographical location for regional, inter-state and international trade with a very high number of truck drivers moving through the area.

Unprotected heterosexual contact has come out to be the most common mode of transmission of HIV in the present study (up to 81.6%).(5) This is in concordance with that reported by Lal *et al*. (84%) in India.(9)

People residing in areas like Jhunjhunu and Sikar are indulged in business activities and usually stay away from their families. This might be the reason behind the very high HIV seropositivity rates from these areas.

Transmission through blood transfusions, once a concern in many countries, has been nearly eliminated in developed countries by the routine screening of blood donations.(10) In developing countries, transmission through the blood supply has yet to be eliminated, especially where HIV prevalence rates among blood donors are high and where screening of blood for HIV has not become routine.(10) India still has many paid blood donors; contaminated blood and blood products account for about 2% of HIV infections.(1) In our region, although screening for HIV before transfusion has been made mandatory since many years, still, around 46 patients gave the history of previous blood transfusion over the study period. Moreover, it is also not easy to judge the route of infection retrospectively by clinical history only.

Injection drug use (IDU) plays a critical role in the HIV epidemic in various regions, particularly Asia and Southern Europe. According to studies in Southeast Asia, HIV prevalence among IDUs rose to 40% within 1 to 2 years after the first positive HIV test result. This was true for Manipur and in North-East India,(10) where intravenous drug abuse is common but not for states like Rajasthan, as clearly shown by this study (only 13 cases fell under this category). Well-documented HIV prevention strategies like needle exchange distribution, bleach distribution, outreach to IDUs, peer education programs, and social network interventions, that have reduced HIV transmission among IDUs in developed countries are now being adopted in developing countries also.(10)

About 16.66% (5/30) eunuchs were also found seropositive in the present study. This may be attributed to the male counselor out reaching the high-risk groups.

Future trends for next couple of years depict further increase in prevalence without any plateau [Figure 3]; this can be useful for State AIDS Control Society (SACS) in planning and arranging resources to deal with the future situation.

## Conclusion

As of now, the care seeking behavior of common people are influenced by so many factors; and it is observed that in case of sensitive issues linked with social stigma like leprosy, STDs, HIV/AIDS there is under reporting and under utilization of facilities. So the probability of different types of pictures in the community setting might not be impossible. A great deal has been learnt about the biological, behavioral, and environmental factors that influence HIV transmission and disease. However, a great deal still remains to be done to translate knowledge into action. It is critical to sustain current HIV prevention and care efforts so that further ground is not lost. However, it is also imperative that we work to expand these efforts, reaching individuals and communities up to the grass root level with prevention and care programs that have been proven to work.
